# White Spots: Prevention in Orthodontics—Systematic Review of the Literature

**DOI:** 10.3390/ijerph20085608

**Published:** 2023-04-21

**Authors:** Assunta Patano, Giuseppina Malcangi, Roberta Sardano, Antonio Mastrodonato, Grazia Garofoli, Antonio Mancini, Alessio Danilo Inchingolo, Daniela Di Venere, Francesco Inchingolo, Gianna Dipalma, Angelo Michele Inchingolo

**Affiliations:** Department of Interdisciplinary Medicine, University of Bari “Aldo Moro”, 70124 Bari, Italy; assuntapatano@gmail.com (A.P.); giuseppinamalcangi@libero.it (G.M.); robertasardano@gmail.com (R.S.); a.mastrodonato14@studenti.uniba.it (A.M.); graziagarofoli.g@libero.it (G.G.); dr.antonio.mancini@gmail.com (A.M.); ad.inchingolo@libero.it (A.D.I.); daniela.divenere@uniba.it (D.D.V.)

**Keywords:** white spots, fixed orthodontic, prevention, demineralization, treatment, fluoroprophylaxis, oral hygiene, fluoride, laser CO_2_, enamel

## Abstract

Early-stage dental demineralization, called white spots (WS), get their name from the characteristic colour that enamel takes on due to the acid attack of salivary cariogenic bacteria. They are often associated with fixed orthodontic therapy (FOT) and, if left untreated, evolve into caries with repercussions on oral health and dental aesthetics. This review aims to identify the most effective prophylaxis strategies to prevent WS during FOT. The search for the reviewed studies was conducted on the Pubmed, Scopus, and Web of Science databases, selecting English-only articles published in the 5 years from January 2018 to January 2023. The keywords used were “WS” and “fixed orthodontic*”, using “AND” as the Boolean operator. A total of 16 studies were included for qualitative analysis. Prevention begins with maintaining proper oral hygiene; fluoride in toothpaste, mouthwashes, gels, varnishes, and sealants can be added to prophylaxis and used regularly. Using a laser in combination with fluoride helps prevent the occurrence of WS and assists in the repair processes of initial lesions. Further studies are needed to establish international guidelines for preventing WS in orthodontically treated patients.

## 1. Introduction

**Figure 1:**
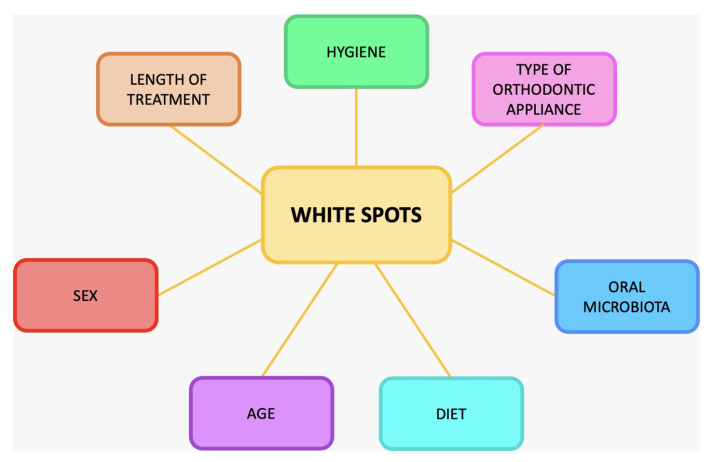
Causes of white spots.

White spots (WS) frequently occur during orthodontic therapy with fixed appliances [1,2,3,4,5]. They usually appear at the gingival and buccal parts of teeth. The teeth most affected by these lesions are the canines and the upper lateral incisors [6,7]. In these areas, losses of enamel mineralization have been determined, which clinically present as more or less extensive areas that are chalky white or brown, porous, and rough to the touch, a phenomenon related to the different diffusion of light compared to normally mineralized enamel [8]. These irreversible lesions of the enamel, if left untreated, evolve into caries [9,10]. The incidence of WS is strictly related to oral hygiene maneuvers and should always be detected by orthodontists at early stages. The recent pandemic situation could have reduced the capability to manage these clinical situations due to the reduced number of appointments [11]. In cases of extended WS or decay, there is a need to perform esthetic restorations; modern restorative materials have several colours and various translucency qualities, allowing them to mirror the optical behaviour of teeth and provide a natural appearance [12]. Their impact on patients’ oral health and smile aesthetics can be very important, hence the importance of WS prevention, which is mainly based on the right selection of patient candidate for orthodontics. The patient in need of orthodontic therapy should first be educated in the most proper home oral hygiene techniques, should acquire a good level of hygiene before even starting orthodontic therapy, and should know that the orthodontic device will hinder common hygiene manoeuvers by representing a receptacle for plaque and bacteria [13]. In addition to hygiene, other factors associated with the occurrence of WS include: sex, age, length of therapy, type of treatment [14], characteristics of the oral bacterial flora, diet followed by the patient, and changes in the microbiota of his or her mouth, all of which have been analyzed in several studies [15,16,17] (Figure 1).

## 2. Materials and Methods

### 2.1. Protocol and Registration

This systematic review was conducted according to the standards of Preferred Reporting Items for Systematic Reviews and Meta-analysis (PRISMA) [18]. The present systematic review has been performed in accordance with the principles of PRISMA and the International Prospective Register of Systematic Review Registry guidelines (ID 405569).

### 2.2. Search Processing

The keywords used in the databases (Scopus, Web of Science, and Pubmed) for the selection of the publications under review were “White Spots” and “fixed orthodont*”, using the word “AND” as the Boolean operator.

The search focused exclusively on articles published in English in the past 5 years (January 2018–January 2023) (Table 1).

### 2.3. Eligibility Criteria

The reviewers worked in pairs, identifying work that met the following inclusion criteria: (1) studies performed only on human subjects; (2) clinical studies or case reports; (3) studies performed on subjects who were in orthodontic therapy (fixed therapy); and (4) studies regarding WS prophylaxis in subjects who were in orthodontic therapy (fixed therapy).

Exclusion criteria were: (1) studies involving therapy of WS after orthodontic therapy; (2) studies involving cure of WS unrelated to orthodontics; (3) in vitro studies; (4) animal studies; (5) systematic reviews, narrative reviews, and meta-analyses.

### 2.4. Data Processing

The screening process, which was conducted by reading the titles and abstracts of the articles selected in the previous identification phase, has allowed excluding all those publications that deviated from the topics examined. Subsequently, the full texts of publications deemed to meet the agreed inclusion criteria, were read. Disagreements between reviewers on article selection were discussed and resolved.

## 3. Results

**Figure 2:**
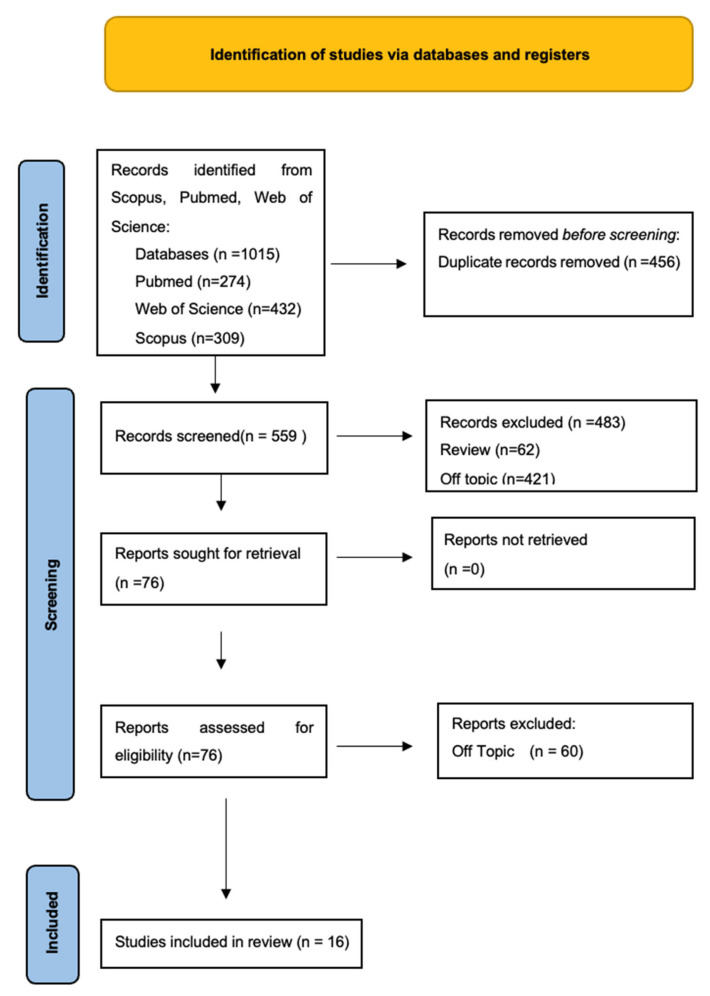
PRISMA flowchart.

Keyword searches of the Web of Science (432), Scopus (309), and Pubmed (274) databases yielded a total of 1015 articles. The subsequent elimination of duplicates (456) resulted in the inclusion of 559 articles. Of these 559 studies, 483 were excluded—62 because they were review and 421 because they were off topic. The writers successfully sought the remaining 76 papers for retrieval, and evaluated their eligibility. The eligibility phase ended with the inclusion of 16 publications for this work (Figure 2). Results of each study were reported in Table 2. The excluded articles have been reported in the Appendix A (Table A1).

## 4. Discussion

Among the most well-known and scientifically validated preventive measures is the use of fluoride in toothpastes, mouthwashes, varnishes, mousses, and cements for bonding brackets and other fixed orthodontic devices [35]. Some strategies, such as antimicrobial toothpastes, amorphous calcium casein phosphopeptides, sealants, lasers, and the presence of antimicrobial substances in orthodontic biomaterials, can effectively prevent WSL in orthodontics [36]. The purpose of this work is to investigate the possible roles of fluoroprophylaxis and other preventive strategies which can help patients and clinicians reduce the occurrence of WS during orthodontic therapy [24,26,37]. 

Fixed orthodontics can have negative repercussions on oral health, as they make home oral hygiene manoeuvres more difficult and are receptacles for bacteria and food debris. This is associated with a higher incidence of WS, caries, and periodontal problems.

### 4.1. Fixed Orthodontics and Salivary Changes

In a clinical study published in 2019 by Jurela et al., 83 patients with a medium age of 15.14 ± 1.66 (52 men and 31 women) receiving FOT were examined [22]. The study’s goal was to estimate the patients’ clinical and salivary changes and see whether there were statistically meaningful variations concerning the type of braces they wore (conventional vs. self-ligating brackets) [21,38].

The DMFT index is the most common population-based measure of caries experience. This index evaluates the total of a person’s decaying, missing, and filled permanent teeth or surfaces. It was considered at the beginning and after six months of orthodontic treatment.

The consequences of treatment on salivary flow, the aspects of WS, and the plaque index were also considered.

Six months following the start of therapy, the study discovered an important rise in DMFT index and salivary flow in all patients, without discrimination depending on the type of fixed appliance utilized (different types of brackets or ligatures). The considerable drop in salivary pH and rise in plaque index may be one cause of the rise in DMFT index. Because increased salivary flow is associated with a rise of the plaque index, which reduces pH, it does not seem to be good to reduce the possibility of carious lesion occurrence [28,39].

### 4.2. Streptococcus mutants and Lactobacillus

In a 2019 comparative prospective study, Jin et al. examined the evolution of these 2 bacterial species in the saliva of people treated with fixed therapy [25]. At four separate time points—T1 before therapy, T2 3 months after appliance fitting, T3 6 months after fitting, and T4 18 months after fitting—the saliva of 15 patients receiving FOT was examined. *Lactobacillus* increased slightly but not significantly over the 18 months of treatment, while total bacteria remained unchanged. The quantity of *S. mutans* was very different between the two types of brackets, after remaining stable for the first six months and increasing dramatically at T4 (*p <* 0.05) [40]. Patients with conventional brackets had a higher amount of *S. mutans* than did those with self-ligating brackets (*p <* 0.05), who had a stable concentration of *S. mutans* during this period. The levels of sIgA, MPO, and LDH did not modify during orthodontic treatment. There was no link between sIgA and bacterial quantity. In conclusion, *S. mutans* increased significantly in patients wearing traditional braces during the last treatment period, suggesting that WS may develop after prolonged orthodontic therapy [20].

### 4.3. Fixed Orthodontics and Caries

Pinto et al., [20] examined INSO (incidence of active caries lesions) in 135 people aged 10 to 30 years. They were split into 2 groups, the first including 70 people who received no orthodontic therapy (G0), and the second including 65 people who received FOT for one year (G1). The plaque index, gingival, and caries indices were assessed at 0 and one years after treatment. One operator evaluated all teeth for caries, examining both active and inactive and early-stage and cavitated lesions. According to the work, the orthodontically treated group had a statistically higher incidence of active caries than the G0 group. In addition, the G1 group had a statistically greater mean increase in active caries. According to the results of this study, people who received FOT for one year had a significantly higher incidence and growth of active caries lesions than did people who did not receive fixed orthodontic therapy.

### 4.4. Enamel Etching and WS

Enamel etching performed before the location of brackets is also believed to be responsible for the rise in caries in subjects undergoing fixed therapy. The study by Yagci et al., 2019 examined possible distinctions between partial and full etching [23]. This was a double-blind randomized controlled trial of 20 patients with a medium age of 16.75 years, excellent dental hygiene, malocclusion, and fixed orthodontic therapy. Full or partial etching treatment was randomly performed on 40 maxillary arches [41]. Quantitative fluorescence images were taken at the start of orthodontic treatment, three (T1) and six (T2) months later, and at the conclusion of the braces removal phase (T3). Using quantitative light fluorescence software, the presence of WS was assessed before and after drilling, and the results were rated with Student’s *t*-test. The research showed that, in terms of Q and A scores at T2, the group with complete etching significantly outperformed the group with partial etching (*p <* 0.05). At every time point, F scores considerably increased in the TE group, but only at T1 and T3 in the PE group. There were no changes between the TE and PE groups at T3 (*p* > 0.05), though. Regardless of the etching approach, the study indicated that the presence of WS were primarily seen in the upper lateral incisors. Although PE is better during the initial 6 months, in terms of long-term WS creation, there is no distinction between PE and TE [42,43].

### 4.5. Prevention of WS in Orthodontics

During orthodontic treatment and in the post-orthodontic phase to achieve remineralization, numerous strategies are employed to prevent enamel demineralization. Use of casein phosphopeptide-containing products, antibacterial products, and fluoride-containing products are examples. Chlorhexidine is the most widely used antibacterial agent for dental usage because it is highly effective against *Streptococcus mutans*. A study by Shimpo et al., assessed the preventive impact of antimicrobial therapy in addition to fluoride application during FOT [30]. With the addictions of fluoride and professional mechanical teeth cleaning, it has been discovered that tooth surface disinfection therapy also helps WS reduction during FOT.

### 4.6. Prevention with Fluoride 

Several studies have found the utility of fluoride toothpaste in the reduction of WS caused by orthodontic therapy [44,45,46,47].

In a prospective study by Kau et al., with three groups of patients receiving orthodontic care [26], Clinpro 5000 was administered to 35 people, Clinpro Tooth Crème was administered to 32 people, and MI Paste Plus was administered to 33 people in every group. For four months, the chosen product was used two times a day for two minutes. Subjects were examined once each month, for 4 months. At each visit, the Enamel Decalcification Index (EDI) was utilized to calculate the amount of WS per square. Compared to previous research, the usage of Clinpro 5000, Clinpro Crème, and MI paste Plus all had a decreasing effect on WS lesions. Clinpro 5000 slightly outperformed the other two test pastes in relation to effectiveness. The clinical trial conducted in 2019 by Smyth et al. came to similar conclusions [33].

A recently introduced fluoride varnish containing 1.5% ammonium fluoride was considered in a 2019 clinical study by Sonesson et al., who ascertained that regular varnish applications reduced the quantity of WS during fixed therapy [32].

Sealants act as physical barriers to bacterial acids and plaque. While good at preventing WS, sealants do peel off over time, predominantly in the gum area, leaving the enamel exposed to plaque and acid bacteria. Sealants like ProSeal have been proven to totally prevent mineral loss from enamel if they stay on the tooth surface, but the application of the product should be repeated every few months [48].

With the growing attention on the host’s innate defense system, more minimally invasive and human-friendly therapies have been considered, like the use of formulas containing enzymes, probiotics, and plant extracts. Intrinsic defense factors in saliva are the enzymes peroxidase, lysozyme, and lactoferrin. These proteins can limit bacterial or fungal growth, interfere with bacterial glucose uptake or glucose metabolism, and promote bacterial aggregation and elimination [49]. Cheng et al., in a 2019 clinical work, compared the effects between enzyme-containing and conventional toothpastes on orthodontic patients [29]. The prevention of WS and plaque reduction effects among orthodontic patients in the first three months of treatment were not significantly different between enzyme-containing and conventional toothpastes, according to the study. In the first three months of treatment, neither gingival bleeding nor visible plaque among orthodontic patients who used fluoride- and enzyme-containing toothpastes significantly increased. However, the gingival bleeding and visible plaque significantly decreased [50,51].

### 4.7. Active Oxygen-Containing Toothpaste

George et al., in an experiment conducted in 2022, examined how streptococcus mutations and WS responded to toothpaste with active oxygen [34]. Active oxygen toothpaste resulted in a more pronounced reduction of WS than did fluoride toothpaste. Its impact was limited, though. Both toothpaste varieties had minimal effects on WSLs. Toothpaste containing active oxygen is effective in the same manner as toothpaste containing fluoride [52,53].

### 4.8. Prevention with CO_2_ Laser

As a result of removing the organic matrix, improving fluoride absorption, and increasing the binding surface area of ions, including calcium and fluoride, fluoride and laser act synergistically to strengthen enamel resistance to acids.

Fluoride affects the creation of fluorohydroxyapatite crystals, changes demineralization and remineralization, and affects bacterial plaque [54,55,56,57]. 

Mahmoudzadeh et al.’s 2019 RCT aimed to estimate the effect of carbon dioxide (CO_2_) laser on the prophylaxis of WS associated with fixed therapy [24]. In this work, 554 teeth from 95 patients were considered. The 95 patients were divided into 2 groups, at random: the laser group (278 teeth), and the control group (276 teeth from 47 patients). The front teeth of the maxilla in the laser group were made aware of the CO_2_ laser with the following characteristics: wavelength 10.6 m, power 0.4 mw, frequency 5 Hz, diameter 0.2 mm, and pulse time 9 s. An operator applied laser irradiation for 20 s while maintaining a 5 mm distance from the buccal surface and moving back and forth continuously [58]. Similar placebo light exposure took place for the control group. Six months after receiving radiation, patients were brought back in to have the incidence, size, and cruelty of the injuries evaluated. Data were collected twice: immediately after adherence to the attack, and six months later. Better lesions and a decrease in lesion incidences were seen during six months with CO_2_ laser use [59,60]. The laser is believed to cause a chemical change in enamel crystals, removing cavities through remineralization. According to the study’s findings, gingival lesions were not affected by laser irradiation, even though it was effective on the incisal, mesial, and distal regions. Unlike the gingival area, where CO_2_ laser had no noticeable impact, the extent of lesions in the incisal, mesial, and distal regions was drastically reduced after treatment. Additionally, while the mesial and incisal portions of the lesion showed a significant reduction in severity, the gingival and distal regions showed little improvement. In the gingival area, the laser was ineffective, most likely because of changes in the thickness and structure of the enamel. Since gingival regions are frequently affected by WSLs, laser settings at these locations should be modified to aid in the reduction of these lesions. Additionally, better oral hygiene can lower the incidence of gingival lesions (due to increased plaque accumulation) [61].

The study by Belcheva et al., which began in September 2021 and whose follow-up phase will last until September 2023, is intriguing in the line of research on the encouraging effects of lasers [21]. Investigating how fluoride varnish and CO_2_ laser treatment can lessen the frequency, severity, and extent of WS lesions during fixed orthodontic therapy is the goal. An RCT will involve kids between the ages of 12 and 18 who need fixed therapy and are at a high risk of developing cavities. The buccal surfaces of the patient’s upper anterior teeth will receive fluoride therapy alone in one group, and fluoride therapy in addition to bonding orthodontic brackets in the other group. Following radiotherapy, the patients’ conditions will be reevaluated six and twelve months later [62,63].

### 4.9. Primer with Antibacterial

Numerous studies on bonding products containing antibacterial substances exist in the literature, and all have shown encouraging results [64,65,66,67].

The aim of the study by Oz et al., is to clinically evaluate an antibacterial primer containing monomer in the prophylaxis of WS during fixed therapy [28]. The study’s findings demonstrate that there was no discernible difference between the antibacterial monomer-containing primer group and the control group in terms of their capacity to prevent demineralization during orthodontic treatment [68]. Degrazia et al., examined the demineralization and antibacterial properties of an experimental orthodontic adhesive made of triazine and niobium bioglass phosphate (TAT) around attachments placed on enamel surfaces [31]. From the results of this study, the growing of *S. mutans* and total streptococcus were inhibited by the adhesive in the triazine and niobium phosphate-based bioglass, which had an anti-demineralization impact. This product can prevent the loss of enamel minerals.

## 5. Conclusions

WS are a common and equally dreaded complication of fixed orthodontics, as they risk seriously compromising the aesthetic and functional outcomes. WS prophylaxis begins with the correct choice [43] and motivation of the subject to maintain good hygiene. In this regard, good oral hygiene with a fluoride-containing toothpaste is the essential starting point for the effective removal of food scraps and bacterial biofilm that are deposited on teeth and braces. In addition, fluoride administration with mouthwashes for home use as well as gels, varnishes, and sealants for periodic professional use may be considered, depending on the case. The use of lasers as an adjunct to fluoride is a readily available avenue for clinicians, effective in the prevention of demineralization but also in the repair processes of early-stage lesions. The hope is that international guidelines for the use of fluoride products, antibacterial agents, and laser use can be developed in the future. More research is required to establish precise and repeatable protocols for laser use. Countless studies in the literature have evaluated the efficacy of toothpastes and other products containing various substances with antibacterial effects, many of which have yielded encouraging results that merit further study. The orthodontist must always remember that the resolution of malocclusions is a goal that must be pursued hand-in-hand with the achievement and maintenance of the patient’s oral and dental health, and in this sense, it is hoped that caries prevention campaigns will have an ever-increasing prevalence and following.

## Figures and Tables

**Table 1:** Database search indicators.

**Article screening** **Strategy**	**Database: Scopus, Web of Science and Pubmed**
	Keywords: A “WS”; B “fixed orthodont*”
	**Boolean variable: AND**
	**Timespan: 2018–2023**
	**Language: English**

**Table 2:** Studies included in the systematic review.

Authors	Type of Study	Object	Study Design and Timeline	Results
Pilli et al., 2022 [19]	RCT	Comparison of efficiency of one rinse per week with NaF and daily rinse with APF (acid phosphate) in preventing WS associated with FOT.	A total of 90 patients, weekly and daily administration (see subject) and subsequent evaluation with ICDAS (International Caries Detection and Assessment System) and GI (Gingival Index) indices.	For the prevention of WS, daily use of acid phosphate mouthwash is more effective than the once-weekly use of sodium fluoride mouthwash.
Pinto et al., 2020 [20]	Longitudinal study	To assess the incidence of active caries in PT undergoing fixed therapy.	135 patients divided into 2 groups, 1 without treatment and 1 with orthodontic treatment, observed for 1 year.	Individuals in fixed therapy for a period of one year had a higher incidence and increase in active carious lesions than did those without fixed braces.
Belcheva et al., 2022 [21]	RCT	Analyze how well the CO_2_ laser and a fluorine-based varnish work together to prevent WS during fixed therapy.	Children who need fixed therapy between the ages of 12 and 18. The buccal surfaces of the anterior upper teeth of the patients in the first group were treated with the CO_2_ laser in conjunction with fluorotherapy, whereas the patients in the second group were treated with a fluoride-based varnish before having brackets placed. Following up is scheduled for 6 and 12 months.	Using CO_2_ lasers to prevent dental caries has enormous potential.
Jurela et al., 2019 [22]	Clinical trial	The primary objective was to assess patients’ DMFTs wearing various types of brackets and ligatures, before and six months into fixed therapy. Finding out how orthodontic treatment affected these individuals’ plaque indices, salivary pHs, flows, and prevalence of WS, was the secondary goal.	52 women and 31 men out of 83 patients were tracked for 2 years.	Fixed therapy may impact intraoral homeostasis, regardless of the attachment and ligature type, since DMFT (decay-missing-filled teeth) index and salivary flow increased significantly while salivary pH declined significantly in all patient groups.
Yagci et al., 2019 [23]	RCT	Determine whether partial or full etching has an impact on the presence of WS.	At the T0, three (T1), and six (T2) months into orthodontic therapy, as well as when the procedure was complete, fluorescence measurements of the enamel surface were taken (T3).	As time goes on, there is no distinction between PE (partial etching) and TE (total etching), in terms of the formation of WSLs.
Mahmoudzadeh et al., 2019 [24]	RCT	Impact of CO_2_ laser during fixed therapy on profilaxis of WS.	95 patients, aged 12 to 30, have 554 anterior upper teeth. In four areas (gingival, incisal, mesial, and distal), at both the initial period and six months following CO_2_ laser irradiation, the incidence, extent, and gravity of lesions were evaluated.	The incidence of WS seems to be reduced thanks to CO_2_ laser irradiation.
Jing et al., 2019 [25]	Prospective comparative cohort study	To investigate changes in the bacteria *Lactobacillus* and *Streptococcus* (S.) mutans in the saliva of individuals with FOT.	During FOT, 15 patients’ immune responses and inflammatory processes, as well as secretory immunoglobulin A (sIgA), myeloperoxidase (MPO), and lactate dehydrogenase (LDH), were examined.	Patients receiving fixed therapy have been found to have an increase in *S. mutans* bacteria in the oral microbiota.
Kau et al., 2019 [26]	RCT	To establish how some fluoride-containing products (Clinpro 5000, Clinpro Tooth Crème, and MI-Paste Plus) influence the development of WS in patients receiving fixed therapy.	120 individuals were recruited for the 3 prospective groups, each of which consisted of 40 orthodontic patients. During four months, the chosen product was brushed twice daily for two minutes. Four months of monthly evaluations were conducted on the subjects. At each visit, EDI was utilized to calculate the quantity of WS per square inch.	All products effectively prevented WS.
Gizani et al., 2016 [27]	RCT	The aim of the study is to evaluate the impact of the daily intake of probiotic bacteria on the appearance of WS and salivary lactobacilli (LB) and mutans streptococci (SM) counts in patients undergoing orthodontic therapy with fixed appliances.	85 patients were randomly divided into 2 groups. Probiotic tablets containing two strains of *Lactobacillus reuteri* were given to the test group once a day. A similar tablet was provided to the placebo group, but it did not contain live bacteria.	Probiotic supplements have not been shown to reduce the appearance of WS.
Oz et al., 2019 [28]	In vivo study	The aim of the study is to investigate the ability of a primer containing antibacterial monomer on the prevention of WS during fixed treatment.	35 patients with a mean age of 14.4 years were identified for (1) optimal oral hygiene; (2) permanent teeth; (3) no restorations on the buccal sides of the teeth; and (4) absence of evident WS on the enamel surfaces of the buccal teeth. Before gluing the fixed appliances, each tooth was thoroughly cleaned and polished.	Throughout the duration of orthodontic therapy, there was no significant difference between the group using antibacterial monomer primers and the control group, in terms of demineralization reduction. The best way to prevent WS during fixed therapy is still considered to be good oral hygiene.
Cheng et al., 2019 [29]	RCT	Comparison of three types of toothpaste: (1) containing enzymes (amyloglucosidase and glucose oxidase); (2) containing fluoride; and (3) natural, chemical-free toothpastes.	42 orthodontic patients (25 women and 17 men, mean age 22.7 ± 4.2 years), divided into 3 groups and assigned to use 3 different types of toothpastes during the first 3 months of treatment.	There are no substantial differences between enzyme-containing toothpaste and fluoride-containing toothpaste, in preventing WS in the first 3 months of fixed therapy.
Shimpo et al., 2022 [30]	RCT	The aim of the study was to investigate the ability of a disinfectant applied to the tooth surface, together with fluorine, to prevent WS during fixed treatment.	Patients aged 13–35 years, on fixed therapy, at high risk of caries due to high levels of *Streptococcus mutans*, who have not taken antibiotics in the weeks prior to salivary sampling.	Tooth surface disinfection together with fluoride application reduces WS during fixed treatment.
Degrazia et al., 2019 [31]	In situ study	This in-depth study tested an experimental orthodontic adhesive with triazine and niobium phosphate bioglass placed around brackets for its ability to fight bacteria and prevent demineralization.		Adhesion of brackets to enamel with adhesives containing triazine and niobium phosphate inverted glass reduces demineralization and/or contributes to the recovery of the mineral content of the enamel. The item can stop enamel from losing minerals, preventing the development of WS.
Sonesson et al., 2020 [32]	RCT	To determine if a new fluoride varnish recipe (1.5% ammonium fluoride) is effective at preventing WS in teenagers receiving multiple orthodontic brackets.	166 patients were divided into 2 groups. Fluoride varnish was administered to the test group’s brackets every six weeks. Placebo group: fluoride-free paint.	The incidence of WS as an adverse effect of fixed therapy was reduced by routine applications of an ammonium fluoride varnish.
Smythe and Noar 2019 [33]	RCT	Clinpro Tooth Crème, Clinpro 5000, and MI Paste Plus on WS development in pt with fixed appliances is the goal of this study. Casein phosphopeptide-amorphous calcium phosphate is present in MI Paste Plus. The toothpastes Clinpro 5000 and Clinpro Tooth Crème each include sodium fluoride.	Use of one of the three products for four months in each of three groups of patients receiving fixed therapy.	The products tested were all able to prevent WS.
George et al., 2022 [34]	RCT	The objective of this study was to evaluate how well fixed treatment patients’ salivary *Streptococcus mutans* growth was inhibited by active oxygen.	Two groups of patients, one used toothpaste with added active oxygen and the other group used fluoride toothpaste.	There is no discernible difference, in preventing WS, between toothpastes containing fluoride and toothpastes with active oxygen.

## Data Availability

Not applicable.

## Abbreviations

FOT	Fixed orthodontic therapy
PT	Patient
RCT	Randomized Controlled Trial
WS	White spots
WSL	White spot lesion
ICDAS	International Caries Detection and Assessment System
GI	Gingival index
DMFT	Decay-missing-filled index
PE	Partial etching
TE	Total etching

## Appendix A

**Table A1:** Articles Excluded after Eligibility.

Articles Excluded	Reason for Exclusion
Balbinot GS, Marcon N, Sauro S, Luxan SA, Collares FM. Alkyl trimethyl ammonium bromide for the formulation of antibacterial orthodontic resins. Clin Oral Investig. 2022 Dec;26(12):7011-7019. doi: 10.1007/s00784-022-04661-0. Epub 2022 Aug 11. PMID: 35951093.	**IN VITRO**
Choi JH, Jung EH, Lee ES, Jung HI, Kim BI. Anti-biofilm activity of chlorhexidine-releasing elastomerics against dental microcosm biofilms. J Dent. 2022 Jul;122:104153. doi: 10.1016/j.jdent.2022.104153. Epub 2022 May 5. PMID: 35526753.	**IN VITRO**
Welk A, Ratzmann A, Reich M, Krey KF, Schwahn C. Effect of self-assembling peptide P_11_-4 on orthodontic treatment-induced carious lesions. Sci Rep. 2020 Apr 22;10(1):6819. doi: 10.1038/s41598-020-63633-0. PMID: 32321955; PMCID: PMC7176635.	**OFF TOPIC**
Tan A, Çokakoğlu S. Effects of adhesive flash-free brackets on enamel demineralization and periodontal status. Angle Orthod. 2020 May 1;90(3):339-346. doi: 10.2319/80819-518.1. PMID: 33378441; PMCID: PMC8032304.	**IN VITRO**
Xu Y, Sun Y, Liu W, Shi Z, Jin X, Xu J, Pan X, Zhang Z, Fu B, Zhang L. Effects of an orthodontic primer containing amorphous fluorinated calcium phosphate nanoparticles on enamel white spot lesions. J Mech Behav Biomed Mater. 2023 Jan;137:105567. doi: 10.1016/j.jmbbm.2022.105567. Epub 2022 Nov 10. PMID: 36379092.	**IN VITRO**
Rajendran R, Sudhakar V, Rangarajan RS, Chinnasamy A, Vasupradha G, Jeeva JS. Evaluation of Change in Surface Enamel Microhardness in Patients Undergoing Fixed Orthodontic Appliance Therapy—A Randomized Control Trial. J Pharm Bioallied Sci. 2021 Nov;13(Suppl 2):S1106-S1110. doi: 10.4103/jpbs.jpbs_259_21. Epub 2021 Nov 10. PMID: 35017939; PMCID: PMC8687020.	**IN VITRO**
Liu L, Zou M. [Electronic probe analysis of enamel remineralization effect of casein phosphopeptide-amorphous calcium phosphate promoted by different concentrations of fluorine]. Zhonghua Kou Qiang Yi Xue Za Zhi. 2018 Jul 9;53(7):470-474. Chinese. doi: 10.3760/cma.j.issn.1002-0098.2018.07.008. PMID: 29996365.	**OFF TOPIC**
Wang YH, Liu F, Liu HN, Wang QX, Xing WZ, Li ZC. [Impact assessment on enamel remineralization after orthodontic treatment with casein phosphopeptide calcium phosphate complex]. Shanghai Kou Qiang Yi Xue. 2018 Aug;27(4):382-385. Chinese. PMID: 30483705.	**OFF TOPIC**
Atilla AO, Ozturk T, Eruz MM, Yagci A. A comparative assessment of orthodontic treatment outcomes using the quantitative light-induced fluorescence (QLF) method between direct bonding and indirect bonding techniques in adolescents: a single-centre, single-blind randomized controlled trial. Eur J Orthod. 2020 Sep 11;42(4):441-453. doi: 10.1093/ejo/cjz058. PMID: 31375814.	**OFF TOPIC**
Coordes SL, Jost-Brinkmann PG, Präger TM, Bartzela T, Visel D, Jäcker T, Müller-Hartwich R. A comparison of different sealants preventing demineralization around brackets. J Orofac Orthop. 2018 Jan;79(1):49-56. English. doi: 10.1007/s00056-017-0116-y. Epub 2018 Jan 12. PMID: 29330611.	**OFF TOPIC**
Shan D, He Y, Gao M, Liu H, Zhu Y, Liao L, Hadaegh F, Long H, Lai W. A comparison of resin infiltration and microabrasion for postorthodontic white spot lesion. Am J Orthod Dentofacial Orthop. 2021 Oct;160(4):516-522. doi: 10.1016/j.ajodo.2020.04.039. Epub 2021 Jul 31. PMID: 34344556.	**OFF TOPIC**
Yi J, Dai Q, Weir MD, Melo MAS, Lynch CD, Oates TW, Zhang K, Zhao Z, Xu HHK. A nano-CaF_2_-containing orthodontic cement with antibacterial and remineralization capabilities to combat enamel white spot lesions. J Dent. 2019 Oct;89:103172. doi: 10.1016/j.jdent.2019.07.010. Epub 2019 Jul 18. PMID: 31326528.	**OFF TOPIC**
Ibrahim AI, Thompson VP, Deb S. A Novel Etchant System for Orthodontic Bracket Bonding. Sci Rep. 2019 Jul 3;9(1):9579. doi: 10.1038/s41598-019-45980-9. PMID: 31270352; PMCID: PMC6610079.	**OFF TOPIC**
Bakry AS, Abbassy MA, Alharkan HF, Basuhail S, Al-Ghamdi K, Hill R. A Novel Fluoride Containing Bioactive Glass Paste is Capable of Re-Mineralizing Early Caries Lesions. Materials (Basel). 2018 Sep 6;11(9):1636. doi: 10.3390/ma11091636. PMID: 30200640; PMCID: PMC6163288.	**OFF TOPIC**
Erbe C, Hartmann L, Schmidtmann I, Ohlendorf D, Wehrbein H. A novel method quantifying caries following orthodontic treatment. Sci Rep. 2021 Nov 1;11(1):21347. doi: 10.1038/s41598-021-00561-7. PMID: 34725354; PMCID: PMC8560919.	**OFF TOPIC**
Erbe C, Jacobs C, Klukowska M, Timm H, Grender J, Wehrbein H. A randomized clinical trial to evaluate the plaque removal efficacy of an oscillating-rotating toothbrush versus a sonic toothbrush in orthodontic patients using digital imaging analysis of the anterior dentition. Angle Orthod. 2019 May;89(3):385-390. doi: 10.2319/080317-520.1. Epub 2018 Dec 5. PMID: 30516414; PMCID: PMC8117681.	**OFF TOPIC**
Kim H, Yoo KH, Yoon SY, Choi YK, Kim YI. A remineralizing orthodontic etchant that utilizes calcium phosphate ion clusters. Front Bioeng Biotechnol. 2022 Aug 31;10:944869. doi: 10.3389/fbioe.2022.944869. PMID: 36118566; PMCID: PMC9473508.	**OFF TOPIC**
Saito T, Park JH, Bay C. A Survey of Pediatric Dentists on the Treatment Timing and Modalities for White Spot Lesions in the United States. J Clin Pediatr Dent. 2019;43(1):27-33. doi: 10.17796/1053-4625-43.1.6. Epub 2018 Dec 6. PMID: 30520700.	**OFF TOPIC**
Al-Khafaji TJ, Agha B, Alhumadi A, Alhamadi WW, Mills D, Davis GR, Cresswell-Boyes AJ, Fleming PS. An assessment of mineral concentration of dental enamel neighbouring hypothetical orthodontic brackets using X-ray microtomography. J Dent. 2022 Nov;126:104306. doi: 10.1016/j.jdent.2022.104306. Epub 2022 Sep 23. PMID: 36162638.	**OFF TOPIC**
Velliyagounder K, Ardeshna A, Shah S. An In Vivo Study on the Development of Bacterial Microbiome on Clear Orthodontic Retainer. Dent J (Basel). 2022 Dec 16;10(12):239. doi: 10.3390/dj10120239. PMID: 36547055; PMCID: PMC9777160.	**OFF TOPIC**
Nafarrate-Valdez RA, Martínez-Martínez RE, Zaragoza-Contreras EA, Áyala-Herrera JL, Domínguez-Pérez RA, Reyes-López SY, Donohue-Cornejo A, Cuevas-González JC, Loyola-Rodríguez JP, Espinosa-Cristóbal LF. Anti-Adherence and Antimicrobial Activities of Silver Nanoparticles against Serotypes *C* and *K* of *Streptococcus mutans* on Orthodontic Appliances. Medicina (Kaunas). 2022 Jun 30;58(7):877. doi: 10.3390/medicina58070877. PMID: 35888596; PMCID: PMC9323808.	**OFF TOPIC**
Sharon E, Sharabi R, Eden A, Zabrovsky A, Ben-Gal G, Sharon E, Pietrokovski Y, Houri-Haddad Y, Beyth N. Antibacterial Activity of Orthodontic Cement Containing Quaternary Ammonium Polyethylenimine Nanoparticles Adjacent to Orthodontic Brackets. Int J Environ Res Public Health. 2018 Mar 27;15(4):606. doi: 10.3390/ijerph15040606. PMID: 29584643; PMCID: PMC5923648.	**OFF TOPIC**
Ferreira CJ, Leitune VCB, Balbinot GS, Degrazia FW, Arakelyan M, Sauro S, Mezzomo Collares F. Antibacterial and Remineralizing Fillers in Experimental Orthodontic Adhesives. Materials (Basel). 2019 Feb 21;12(4):652. doi: 10.3390/ma12040652. PMID: 30795577; PMCID: PMC6416618.	**OFF TOPIC**
Denis H, Werth R, Greuling A, Schwestka-Polly R, Stiesch M, Meyer-Kobbe V, Doll K. Antibacterial properties and abrasion-stability: Development of a novel silver-compound material for orthodontic bracket application. J Orofac Orthop. 2022 Jul 18. English. doi: 10.1007/s00056-022-00405-7. Epub ahead of print. PMID: 35849137.	**OFF TOPIC**
Yan J, Yang H, Luo T, Hua F, He H. Application of Amorphous Calcium Phosphate Agents in the Prevention and Treatment of Enamel Demineralization. Front Bioeng Biotechnol. 2022 May 13;10:853436. doi: 10.3389/fbioe.2022.853436. PMID: 35646855; PMCID: PMC9136455.	**OFF TOPIC**
Mahmood HT, Kamal AT, Khan BN, Fida M. Application of New Biomedical Materials in Orthodontic Appliances. J Coll Physicians Surg Pak. 2019 Jul;29(7):654-657. doi: 10.29271/jcpsp.2019.07.654. PMID: 31253218.	**OFF TOPIC**
Schneider BJ, Hiers RD, Currier GF, Kadioglu O, Johnston SE, Zhao YD, Esteban Florez FL, Khajotia SS. Assessment of *Streptococcus mutans* biofilms on orthodontic adhesives over 7 days. Am J Orthod Dentofacial Orthop. 2021 Jul;160(1):50-57. doi: 10.1016/j.ajodo.2020.03.026. Epub 2021 Jun 3. PMID: 34090735; PMCID: PMC8238838.	**OFF TOPIC**
Gulec A, Goymen M. Assessment of the resin infiltration and CPP-ACP applications before orthodontic brackets bonding. Dent Mater J. 2019 Oct 2;38(5):854-860. doi: 10.4012/dmj.2019-021. Epub 2019 Aug 22. PMID: 31434834.	**OFF TOPIC**
Al-Eesa NA, Karpukhina N, Hill RG, Johal A, Wong FSL. Bioactive glass composite for orthodontic adhesives—Formation and characterisation of apatites using MAS-NMR and SEM. Dent Mater. 2019 Apr;35(4):597-605. doi: 10.1016/j.dental.2019.02.010. Epub 2019 Feb 23. PMID: 30808559.	**OFF TOPIC**
Poornima P, Krithikadatta J, Ponraj RR, Velmurugan N, Kishen A. Biofilm formation following chitosan-based varnish or chlorhexidine-fluoride varnish application in patients undergoing fixed orthodontic treatment: a double blinded randomised controlled trial. BMC Oral Health. 2021 Sep 23;21(1):465. doi: 10.1186/s12903-021-01805-8. PMID: 34556107; PMCID: PMC8459499.	**OFF TOPIC**
Marques Ferreira de Sena L, Monielle Duarte Moura D, Helena Gurgel de Carvalho I, de Fatima Dantas de Almeida L, Ramos da Silva N, Othávio de Assunção E Souza R. Bond strength, degree of conversion, and microorganism adhesion using different bracket-to-enamel bonding protocols. J Orofac Orthop. 2022 Oct 17. English. doi: 10.1007/s00056-022-00430-6. Epub ahead of print. PMID: 36251054.	**OFF TOPIC**
Yang H, Ma Y, Xie X, Wang H, Li X, Fang D, Bai Y. Candida albicans enriched in orthodontic derived white spot lesions and shaped focal supragingival bacteriome. Front Microbiol. 2023 Jan 24;14:1084850. doi: 10.3389/fmicb.2023.1084850. PMID: 36760510; PMCID: PMC9902512.	**OFF TOPIC**
Philip N, Leishman SJ, Bandara HMHN, Walsh LJ. Casein Phosphopeptide-Amorphous Calcium Phosphate Attenuates Virulence and Modulates Microbial Ecology of Saliva-Derived Polymicrobial Biofilms. Caries Res. 2019;53(6):643-649. doi: 10.1159/000499869. Epub 2019 Jun 4. PMID: 31163430.	**OFF TOPIC**
Comert S, Oz AA. Clinical effect of a fluoride-releasing and rechargeable primer in reducing white spot lesions during orthodontic treatment. Am J Orthod Dentofacial Orthop. 2020 Jan;157(1):67-72. doi: 10.1016/j.ajodo.2019.06.013. PMID: 31901283.	**OFF TOPIC**
Kannan A, Padmanabhan S. Comparative evaluation of Icon^®^ resin infiltration and Clinpro™ XT varnish on colour and fluorescence changes of white spot lesions: a randomized controlled trial. Prog Orthod. 2019 Jun 17;20(1):23. doi: 10.1186/s40510-019-0276-y. Erratum in: Prog Orthod. 2019 Jul 26;20(1):31. PMID: 31204437; PMCID: PMC6571438.	**OFF TOPIC**
Korkmaz YN, Yagci A. Comparing the effects of three different fluoride-releasing agents on white spot lesion prevention in patients treated with full coverage rapid maxillary expanders. Clin Oral Investig. 2019 Aug;23(8):3275-3285. doi: 10.1007/s00784-018-2749-7. Epub 2018 Nov 28. PMID: 30488120.	**OFF TOPIC**
Küçükönder A, Hatipoğlu Ö. Comparison between a glass ionomer cement and a compomer concerning bonded acrylic expander retention and white spot formation: A randomized clinical trial. J Orofac Orthop. 2023 Feb 10. English. doi: 10.1007/s00056-023-00448-4. Epub ahead of print. PMID: 36764948.	**OFF TOPIC**
Meyer-Kobbe V, Doll K, Stiesch M, Schwestka-Polly R, Demling A. Comparison of intraoral biofilm reduction on silver-coated and silver ion-implanted stainless steel bracket material: Biofilm reduction on silver ion-implanted bracket material. J Orofac Orthop. 2019 Jan;80(1):32-43. doi: 10.1007/s00056-018-00165-3. Epub 2018 Dec 10. PMID: 30535568; PMCID: PMC6334737.	**OFF TOPIC**
Knösel M, Vogel Alvarez R, Blanck-Lubarsch M, Helms HJ. Comparison of potential long-term costs for preventive dentistry treatment of post-orthodontic labial versus lingual enamel cavitations and esthetically relevant white-spot lesions: a simulation study with different scenarios. Head Face Med. 2019 Aug 9;15(1):22. doi: 10.1186/s13005-019-0204-x. PMID: 31399113; PMCID: PMC6688377.	**OFF TOPIC**
Knaup I, Kobbe C, Ehrlich EE, Esteves-Oliveira M, Abou-Ayash B, Meyer-Lueckel H, Wolf M, Wierichs RJ. Correlation of quantitative light-induced fluorescence and qualitative visual rating in infiltrated post-orthodontic white spot lesions. Eur J Orthod. 2023 Mar 31;45(2):133-141. doi: 10.1093/ejo/cjac051. PMID: 36179095.	**OFF TOPIC**
Wang Y, Hua F, Jiang H. CPP-ACP May be effective, but not Significantly Greater than using Fluorides Alone, in Preventing and Treating white Spot Lesions Around Orthodontic Brackets. J Evid Based Dent Pract. 2020 Mar;20(1):101416. doi: 10.1016/j.jebdp.2020.101416. Epub 2020 Feb 20. PMID: 32381413.	**OFF TOPIC**
Hua F, Yang H, He H. Current Enamel Remineralization Therapies Have Limited Effects on Postorthodontic White Spot Lesions. J Evid Based Dent Pract. 2018 Dec;18(4):339-342. doi: 10.1016/j.jebdp.2018.10.002. Epub 2018 Oct 16. PMID: 30514448.	**OFF TOPIC**
Cardoso AA, de Sousa ET, Steiner-Oliveira C, Nobre-Dos-Santos M. Debonding of orthodontic appliance changes salivary physicochemical properties and favors regression of active caries lesions: A 13-week follow-up study. Int J Paediatr Dent. 2022 Jul;32(4):607-616. doi: 10.1111/ipd.12939. Epub 2022 Feb 13. PMID: 34779541.	**OFF TOPIC**
Şen S, Erber R, Deurer N, Orhan G, Lux CJ, Zingler S. Demineralization detection in orthodontics using an ophthalmic optical coherence tomography device equipped with a multicolor fluorescence module. Clin Oral Investig. 2020 Aug;24(8):2579-2590. doi: 10.1007/s00784-019-03116-3. Epub 2019 Dec 17. PMID: 31848715.	**OFF TOPIC**
Umeh OD, Utomi IL, Ndukwe AN, Izuka M. Demineralization preventive practices among Nigerian orthodontists-An evidence-based approach? Niger J Clin Pract. 2020 May;23(5):589-595. doi: 10.4103/njcp.njcp_315_19. PMID: 32367863.	**OFF TOPIC**
Askar H, Krois J, Rohrer C, Mertens S, Elhennawy K, Ottolenghi L, Mazur M, Paris S, Schwendicke F. Detecting white spot lesions on dental photography using deep learning: A pilot study. J Dent. 2021 Apr;107:103615. doi: 10.1016/j.jdent.2021.103615. Epub 2021 Feb 19. PMID: 33617941.	**OFF TOPIC**
Jia A, Wang P, Tong F, Chen Z, Deng Y, Yao H, Wang L, Liu Y, Ge H. Developing a Novel Enamel Adhesive with Amorphous Calcium Phosphate and Silver Nanoparticles to Prevent Demineralization during Orthodontic Treatment. J Funct Biomater. 2023 Jan 29;14(2):77. doi: 10.3390/jfb14020077. PMID: 36826876; PMCID: PMC9966906.	**OFF TOPIC**
Sampson V, Sampson A. Diagnosis and treatment options for anterior white spot lesions. Br Dent J. 2020 Sep;229(6):348-352. doi: 10.1038/s41415-020-2057-x. Epub 2020 Sep 25. PMID: 32978577.	**REVIEW**
Yetkiner E, Gürlek Ö, Işık A, Lappin DF, Buduneli N. Do Adhesive Flash-free Brackets Affect Bacterial Plaque in Patients with Adequate Oral Hygiene? A Randomised Controlled Clinical and Microbiological Assessment. Oral Health Prev Dent. 2019;17(6):533-539. doi: 10.3290/j.ohpd.a43753. PMID: 31825025.	**OFF TOPIC**
Rafiei E, Fadaei Tehrani P, Yassaei S, Haerian A. Effect of CO_2_ laser (10.6 μm) and Remin Pro on microhardness of enamel white spot lesions. Lasers Med Sci. 2020 Jul;35(5):1193-1203. doi: 10.1007/s10103-020-02970-y. Epub 2020 Feb 1. PMID: 32006264.	**OFF TOPIC**
Yetkin D, Sayar G. Effect of Fluoride Releasing Bonding Materials on Shear Bond Strength of Orthodontic Brackets. Turk J Orthod. 2020 Mar 1;33(1):52-58. doi: 10.5152/TurkJOrthod.2020.19052. PMID: 32284899; PMCID: PMC7138233.	**OFF TOPIC**
Salamara O, Papadimitriou A, Mortensen D, Twetman S, Koletsi D, Gizani S. Effect of fluoride varnish with functionalized tri-calcium phosphate on post-orthodontic white spot lesions: an investigator-blinded controlled trial. Quintessence Int. 2020;51(10):854-862. doi: 10.3290/j.qi.a44810. PMID: 32577707.	**OFF TOPIC**
Ferreira RS, Ricomini-Filho AP, Tabchoury CP, Vale GC. Effect of high-fluoride dentifrice and bracket bonding composite material on enamel demineralization in situ. Clin Oral Investig. 2020 Sep;24(9):3105-3112. doi: 10.1007/s00784-019-03182-7. Epub 2020 Jan 2. PMID: 31897706.	**OFF TOPIC**
Ali A, Ismail H, Amin K. Effect of nanosilver mouthwash on prevention of white spot lesions in patients undergoing fixed orthodontic treatment—a randomized double-blind clinical trial. J Dent Sci. 2022 Jan;17(1):249-255. doi: 10.1016/j.jds.2021.03.016. Epub 2021 May 1. PMID: 35028045; PMCID: PMC8739266.	**OFF TOPIC**
Ghadirian H, Geramy A, Shallal W, Heidari S, Noshiri N, Keshvad MA. The Effect of Remineralizing Agents With/Without CO_2_ Laser Irradiation on Structural and Mechanical Properties of Enamel and its Shear Bond Strength to Orthodontic Brackets. J Lasers Med Sci. 2020 Spring;11(2):144-152. doi: 10.34172/jlms.2020.25. Epub 2020 Mar 15. PMID: 32273955; PMCID: PMC7118509.	**OFF TOPIC**
Alqahtani MA, Almosa NA, Alsaif KA, Alsaif NM, Aljaser YJ. Effect of topical fluoride application and diode laser-irradiation on white spot lesions of human enamel. Saudi Dent J. 2021 Dec;33(8):937-943. doi: 10.1016/j.sdentj.2021.08.007. Epub 2021 Aug 23. PMID: 34938035; PMCID: PMC8665184.	**IN VITRO**
Handa A, Chengappa D, Sharma P, Handa JK. Effectiveness of Clinpro Tooth Crème in comparison with MI Varnish with RECALDENT™ for treatment of white spot lesions: a randomized controlled trial. Clin Oral Investig. 2022 Nov 2. doi: 10.1007/s00784-022-04766-6. Epub ahead of print. PMID: 36322154.	**OFF TOPIC**
Yıldırım ZB, Ramoğlu Sİ. Effects of a flash-free system on dental plaque accumulation and bonding-debonding process: A clinical study. Am J Orthod Dentofacial Orthop. 2023 Jan;163(1):54-59. doi: 10.1016/j.ajodo.2021.08.024. Epub 2022 Oct 8. PMID: 36216622.	**OFF TOPIC**
Hennig CL, Blochberger B, Symmank J, Nitzsche Á, Nietzsche S, Steiniger F, Dederichs M, Güllmar A, Reise M, Schulze-Späte U, Sigusch B, Jacobs C. Effects of reducing excess dental adhesive on bacterial adhesion in the bracket periphery. Clin Oral Investig. 2023 Feb 21. doi: 10.1007/s00784-023-04924-4. Epub ahead of print. PMID: 36809356.	**OFF TOPIC**
Gómez C, Abellán R, Palma JC. Efficacy of photodynamic therapy vs ultrasonic scaler for preventing gingival inflammation and white spot lesions during orthodontic treatment. Photodiagnosis Photodyn Ther. 2018 Dec;24:377-383. doi: 10.1016/j.pdpdt.2018.11.001. Epub 2018 Nov 3. PMID: 30399455.	**OFF TOPIC**
